# Update on Musculoskeletal Pain Management

**DOI:** 10.1055/s-0043-1776135

**Published:** 2024-02-01

**Authors:** André Wan Wen Tsai, Ricardo Kobayashi, Ibrahim Afrânio Willi Liu, Márcio Fim, André Cicone Liggieri, Edilson Silva Machado

**Affiliations:** 1 Colégio Médico de Acupuntura do Estado de SP, São Paulo, SP, Brasil; 2 Centro de Dor, Hospital das Clínicas, Faculdade de Medicina, Universidade de São Paulo, São Paulo, SP, Brasil; 3 Universidade Federal de Minas Gerais, Belo Horizonte, MG, Brasil; 4 Cirurgia de Ombro e Cotovelo, Instituto de Previdência dos Servidores do Estado de Minas Gerais (IPSEMG), Belo Horizonte, MG, Brasil; 5 Serviço de Dor e Cuidados Paliativos, Hospital Nossa Senhora da Conceição, Porto Alegre, RS, Brasil

**Keywords:** acute pain, chronic pain, musculoskeletal pain, pain management, postoperative pain

## Abstract

Pain is the most common complaint reported to orthopedists in the outpatient clinic, emergency room, or booth. Numerous publications report the inadequate management of both acute and chronic pain by health professionals. This updated article aims to provide information about musculoskeletal pain, its classification, evaluation, diagnosis, and the multimodal therapeutic approach for each case. For acute pain, adequate control allows for earlier rehabilitation to work and reduces the rates of pain chronification. For chronic pain, the goal is to reduce its intensity and improve the quality of life. Currently, some procedures are increasingly used and aided by imaging tests for diagnostic and therapeutic purposes.

## Introduction

One in every five adults in the world experiences pain. Pain is the chief complaint leading subjects to seek medical services at an outpatient and emergency level. It results in disability on an individual level and a high economic impact on society. 1 2

According to the International Association for the Study of Pain (IAPS), pain is an unpleasant sensory and emotional experience resulting from actual or potential tissue damage or similarly associated with it. 3 Pain classification relies on time (acute or chronic) and its pathophysiology (nociceptive, neuropathic, nociplastic, or mixed). The principles for treating cancer and non-cancer pain are different. Therefore, this article discusses the assessment and management of non-cancer musculoskeletal pain.

## Pathophysiology of Pain

Pain is an experience perceived by the central nervous system. This perception requires information to reach the brain through neuronal pathways and circuits. Thus, pain perception by the somatosensory cortex begins in the periphery. The painful stimulus transforms into mechanical, thermal, or chemical stimuli through neuronal signaling ( *transduction* ). These stimuli, in turn, are preferentially relied on by type A-delta or C neural ( *transmission* ) for the first synaptic connection with neurons from the posterior horn of the spinal cord (PHSC). In PHSC, inhibitory and excitatory stimuli ( *modulation* ) from the peripheral and central nervous system influence pain information (descending inhibitory system). 4

To understand this pathophysiology, we must define nociception as the perception of a painful stimulus depending on the integrity of the previously mentioned pathways. In contrast to nociception, pain is a complex experience involving several neuronal modulation phenomena. 5

Patients often present painful complaints with no correlation with complementary tests despite the lack of structural changes in imaging. The justification for their complaints may lie in a phenomenon known as sensitization, defined as an increase in the excitability of the cell membrane of sensory neurons in the face of painful stimuli, which may be peripheral (nerve ending) or central (PHSC and the entire neuroaxis). 6

## Epidemiology

Chronic pain prevalence rates range from 11% to 40% in the American population (average, 20.4%). In the United Kingdom, the prevalence of chronic pain is 43.5%, and the rate of moderate to disabling pain ranges from 10.4% to 14.3%. Brazilian data indicate that the prevalence of chronic pain ranges from 23.02 to 76.17% (average rate, 45.59%), affecting more females. The highest prevalence in Brazil occurs in the Midwest region (56.25%). Regarding the IASP mechanism classification, nociceptive, neuropathic, and nociplastic pain prevalence is 36.70%, 14.5%, and 12.5%, respectively. 7

## Pain Classification

**Fig. 1:**
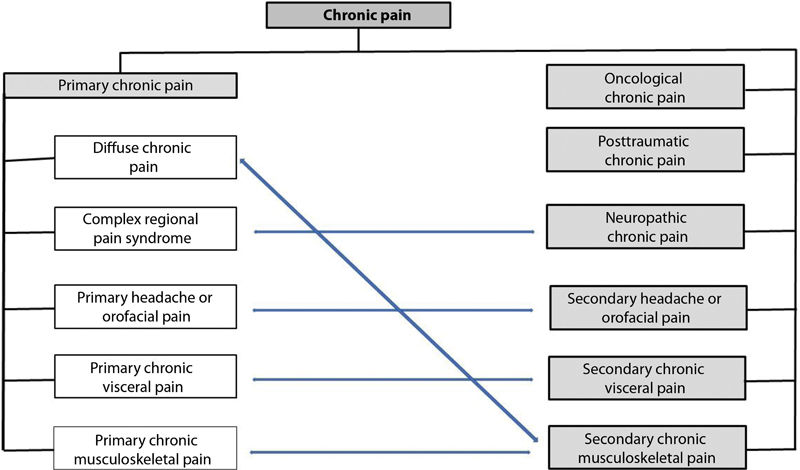
CID-11 classification of chronic pain. 11

**Fig. 2:**
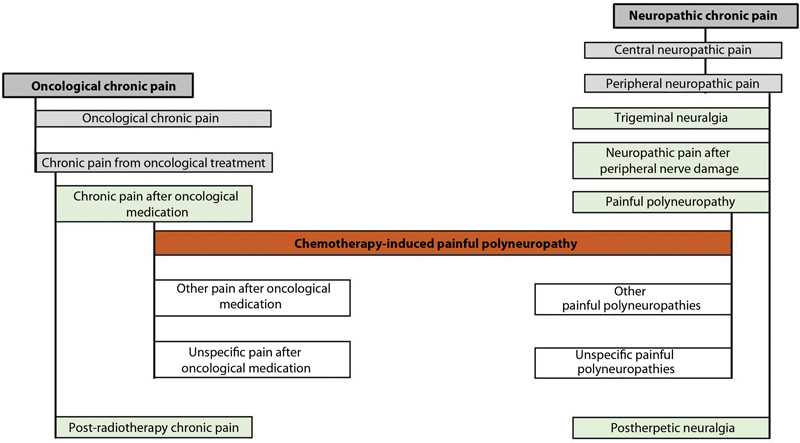
Subdivision of chronic pain syndromes per CID-11. 11

The correct identification of the type of pain is essential for the appropriate therapeutic choice since each has a different treatment. 8 IASP reformulated the international classification of diseases (ICD- 11) to favor research and diagnoses and improve the interpretation of several chronic pain syndromes. Fig. 1 and 2 show the new division. 9

## Regarding Evolution Time

Acute pain is a normal physiological response of the body to painful stimuli. It is a self-limited and short-lasting condition, often for less than three months, ceasing at the end of affected tissue healing and repair. 8 10

Chronic pain persists beyond the regular healing time or more than three months and presents all the repercussions potentially associated with it.

## Regarding the Type of Pain

**Fig. 3:**
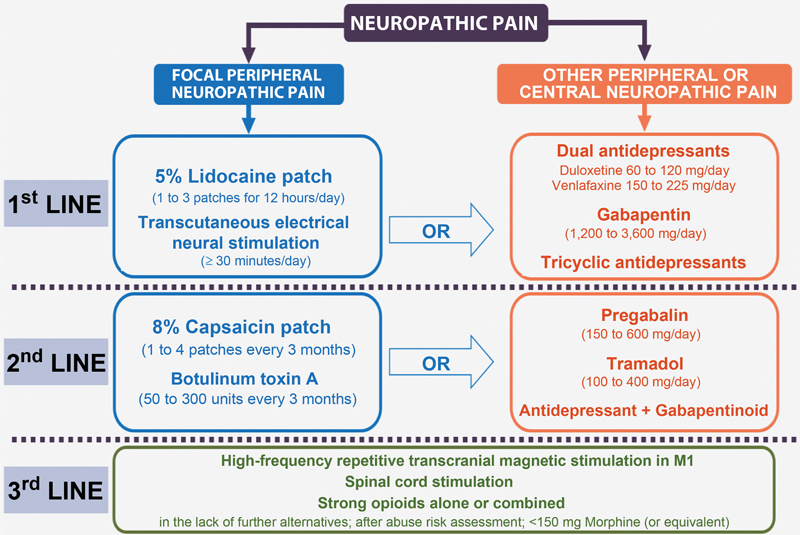
Guidelines from the 2020 French recommendations for neuropathic pain treatment. 17

- Nociceptive pain arises from actual or potential damage to non-neural tissues. 11 It occurs in acute traumatic and postoperative pain. The most commonly used medication classes for its treatment are simple or opioid analgesic agents, non-steroidal anti-inflammatory drugs (NSAIDs), and muscle relaxants ( Table 1 ). 6
- Neuropathic pain (NP) results from an injury or dysfunction of peripheral or central nervous system structures, as in disc herniation with root compression and carpal tunnel syndrome ( Table 2 ). 12 The clinical manifestations include burning pain, painful cold, electric shock, itching, and pins and needles sensation. In addition, the neurological examination reveals hyperalgesia, hyperpathy, and allodynia. The leading treatment for this pain consists of adjuvant medications (tricyclic antidepressants, dual antidepressants, and gabapentinoid anticonvulsants). Opioids are reserved for analgesia at the beginning of treatment until the adjustment of the adjuvant dose and to allow rehabilitation when analgesia is insufficient. 8 Please refer to Fig. 3. 12
- Nociplastic pain is the more recently described pain. It has no association with any tissue injury (neural or not). Examples of nociplastic pain include migraine and fibromyalgia. Its treatment relies on multimodal and interdisciplinary approaches. 12
- Mixed pain combines the pain types described above in a single subject. 4

**Table 1:** Main medications recommended for nociceptive pain in adults.

		Initial dose	Posology	Therapeutic dose	Notes
Simple analgesic agents					
Dipyrone (metamizole)		500 mg to 1 g	6/6 hours	Up to 4 g/day	Agranulocytosis risk 24
Paracetamol		500 to 750 mg	6/6 hours	Up to 3 g/day	Hepatotoxicity 23
**Non-steroidal anti-inflammatory drugs: recommended for acute pain in minimal doses and for short courses**					
Non-selective		Variable	Variable	Variable	Gastrointestinal and renal effects 28
Cox-2 selective		Variable	Variable	Variable	Cardiovascular effects 32
***Opioid analgesic agent (per os)***					
Weak	Codeine	15 to 30 mg	Up to 6/6 hours	Variable Maximum dose: 360 mg/day	Dose-dependent: euphoria, nausea, obstipation, addiction, sedation, and respiratory depression 36
	Tramadol	50 to 100 mg	Up to 4/4 hours	Variable Maximum dose: 400 mg/day	
Strong	Morphine	5 to 30 mg	Up to 4/4 hours	Variable No maximum dose	
	Oxycodone	10 mg	12/12 hours	Variable No maximum dose	
	Methadone	2.5 to 5 mg	Up to 4/4 hours	Variable No maximum dose	
	Buprenorphine	5 mg	Every 7 days	Variable No maximum dose	
**Antispasmodic muscle relaxant agents**						
Carisoprodol		350 mg	Up to 6/6 hours	Maximum dose: 1.400 mg/day	Postural hypotension, drowsiness, and dizziness 45	
Cyclobenzaprine		5 mg a 10 mg	Up to 8/8 hours	20 to 40 mg Maximum dose: 60 mg/day	Drowsiness, dizziness, and dry mouth 46	

**Table 2:** First-line drugs for neuropathic pain treatment according the main guidelines 49

		Initial dose	Posology	Therapeutic dose	Adverse effects
***Antidepressant agents***					
Tricyclic	Amitriptyline Imipramine Nortriptyline	10 to 25 mg	Once per day, 3 hours before sleeping	25 to 150 mg/day	Drowsiness, dizziness, tremors, headaches, postural hypotension, dry mouth, nausea, constipation, weight gain, decreased libido, hyperhidrosis, urinary retention, and suicidal ideation 47
Dual	Duloxetine	30 mg	Once a day, in the morning	60 to 120 mg/day	Nausea, dry mouth, drowsiness, increased blood pressure, and suicidal ideation 48
	Venlafaxine	37.5 to 75 mg	Once a day, in the morning	150 to 225 mg/day	
***Anticonvulsant agents***					
Gabapentin (GBP)		300 mg	8/8 hours	1,200 to 3,600 mg/day	Dizziness, nausea, drowsiness, edema of extremities, weight gain, blurred vision, and suicidal ideation 48
Pregabalin (PGL)		50 to 75 mg	12/12 hours	150 to 600 mg/day	
*Topical*					
5% lidocaine patch		1 to 3 patches	12 hours without the patch/12 hours with the patch	1 to 3 patches	

### Pain Assessment and Diagnosis

Patient assessment must be complete, with anamnesis, physical examination, and subsidiary tests. It is critical to determine when, how, and any factors improving or worsening pain, and the previously prescribed treatments and their outcomes. It is also fundamental to describe the actual definition of whether or not a clinical-radiological correlation can establish a causal relationship to pain. Some patients, especially those with chronic pain, already tried many treatments, and several life dimensions have been affected (quality of life, functionality, social relationships, etc.). Scales can facilitate the subjective measurement of pain (numeric verbal scale and analog verbal scale [VNS, VAS]). Neuropathy pain screening tools may help, including DN4, LANSS, and painDetect. The assessment must clarify the main mechanisms involved in pain and any causal relationship. 13 14 15 16

### Treatment

#### Pharmacological Treatment

**Fig. 4:**
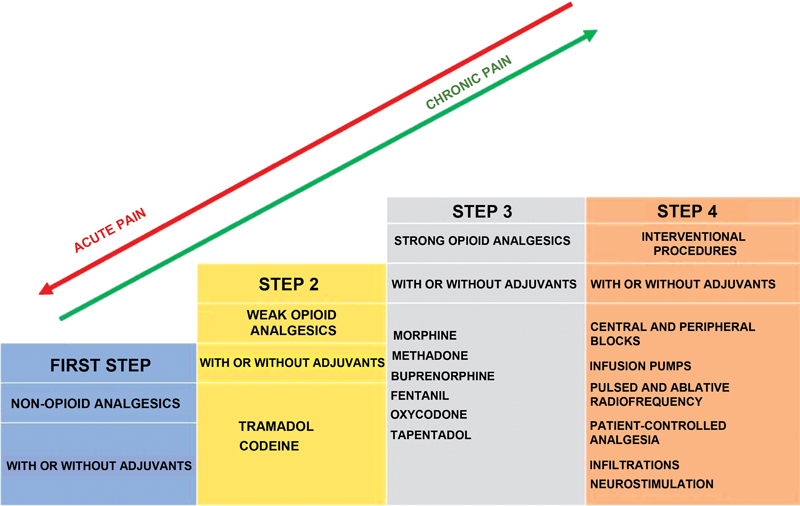
Adaptation of the WHO analgesic ladder for stepped analgesia. Medications in the first step are indicated for mild pain; those in the second step are used for moderate pain, and those in the third step treat severe pain. Later, a fourth step was added to the original ladder to address drug treatment-refractory pain. 22 23 .

Diagnosis and specific causal treatment are essential for acute pain since this is a physiological function of warning of an active injury or disease. Symptomatic pain treatment adopts the World Health Organization (WHO) analgesic ladder 17 per pain intensity (VAS or VNS). The first step of the WHO analgesic ladder ( Fig. 4 ) refers to mild pain, using simple analgesics combined with an NSAID and considering any contraindication to their use. The second step (moderate pain) employs weak opioids. The third step (severe pain) uses potent opioids. An adapted WHO analgesic ladder adds a fourth step, consisting of interventional procedures for pain control. 18

Several factors result in the progression of acute to chronic pain, including pain intensity, social and psychological aspects, and even genetic factors. However, proper treatment of acute pain is essential to prevent its progression. In the orthopedic context, the type of anesthesia and the drugs used in the perioperative period can reduce the risk of chronic pain. 8 Failure in medical training in pain remains a leading cause of inadequate pain management. 8

All chronic pain treatment requires a multimodal approach, using pharmacological and non-pharmacological strategies and different mechanisms of action with synergistic potential. 19

#### Simple Analgesic Agents

Dipyrone (metimazole) and paracetamol are the most used medications in clinical practice. Both are analgesics and antipyretics with similar efficacy and little anti-inflammatory effects by inhibiting cyclooxygenases type 1 (COX-1) and type 2 (COX-2). 20 21 They present different side effect profiles. Paracetamol can be hepatotoxic in high doses and is the leading cause of acute liver failure in the USA. 22 Paracetamol is safe in dosages of up to 4 g/day. In turn, dipyrone may cause agranulocytosis as an adverse effect. Nevertheless, the incidence of this side effect is very low in Latin America. 23 Dipyrone and paracetamol treat acute and chronic pain and do not require dose adjustment in subjects with renal failure. 24

#### Non-steroidal Anti-Inflammatory Agents

NSAID indications include acute pain, chronic pain exacerbation, and inflammatory pain from nociceptive causes. The current recommendation is to use the lowest dose possible for a few days due to the risk of side effects, especially with prolonged treatment. 25

NSAIDs block prostaglandin synthesis by inhibiting the constitutive enzyme COX-1, generating adverse gastrointestinal effects, and COX-2, induced by the inflammatory process. 26 The lower COX-2 selectivity reduces the risk of adverse gastrointestinal events and bleeding, whereas the higher COX-2 selectivity increases the chance of cardiovascular effects. NSAIDs are the most commonly used medications to treat chronic pain, even though there is no recommendation for this indication. They are contraindicated subjects with renal and hepatic insufficiency. 26

#### Opioid Analgesic Agents

**Fig. 5:**
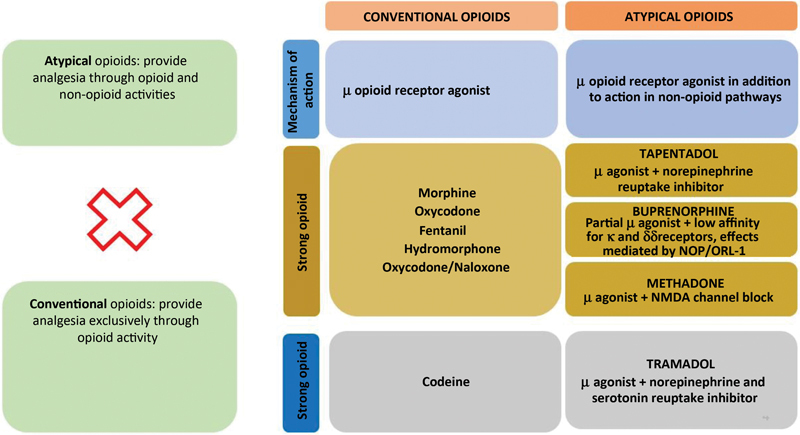
Opioid classification per potency and mechanism of action. 35 36 37 38 39 40 41 49

Opioids can be typical or atypical, weak or strong ( Fig. 5 ). Weak opioids may treat acute moderate or severe pain and chronic pain exacerbations. Strong opioids are precisely indicated in acute and severe pain and eventually for cases of difficult pain control. 27

Despite fears on the part of orthopedists, the rational use of opioids makes prescriptions safe. Adequate titration, research into the risk of addiction, and administration for the shortest possible time until adjuvant medications or specific treatments produce the desired effect are good practice. 28

In practice, use a short-acting opioid such as morphine titrated to the lowest effective dose with successive increases until pain relief. From this dose, adjust rotation calculations or even rescue doses. Rescue uses 1/10 to 1/6 of the total daily dosage for pain between intervals, as well as for controlled release presentations and equianalgesic dose for opioid rotation. Several empirical conversion tables serve as guidance. Cross-tolerance, opioid-induced hyperalgesia, or intolerable effects are criteria for opioid rotation. 29

Other side effects of opioids include constipation, nausea, vomiting, hyperalgesia, tolerance, and withdrawal. Constipation is intolerable and must receive prophylactic treatment, as it increases morbidity and mortality and worsens the quality of life. Constipation treatment may employ laxatives, such as lactulose, bisacodyl, senna, and polyethylene glycol, in addition to dietary changes and hydration. Older subjects warrant special care during opioid use due to the increased risk of adverse effects and falls. 30

Continued medical education is essential for what happened in the United States does not occur in Brazil. 31

### Tramadol

Tramadol is a weak opioid with agonist activity at opioid receptors (μ) and dual action by inhibiting serotonin and norepinephrine reuptake. It helps treat nociceptive and neuropathic pain. 32

Its disadvantage is the decrease in the seizure threshold. Concomitant use with antidepressants, especially selective serotonin reuptake or dual antidepressants, can result in serotoninergic syndrome. It causes less constipation than codeine.

Dose adjustment is required in subjects with advanced renal failure, as it is dialyzable and safe for dialysis patients. 32

### Codeine

Codeine is a morphine derivative undergoing liver metabolization into codeine-6-glucuronide and demethylation into morphine, forming active metabolites acting on opioid receptors (μ). 33 Some patients do not have the codeine-converting enzyme and are insensitive to the medication. As its elimination is renal, it requires care in patients with renal insufficiency. Since codeine removal through dialysis is complicated, it is best to avoid it in dialysis patients. 34

### Morphine

Morphine is a natural opioid with agonist activity at opioid receptors (μ). It is a reference for titration and calculation of equianalgesic doses for opioid rotation. Morphine is available in immediate and controlled-release presentations.

It undergoes hepatic metabolism into morphine-3-glucuronide, morphine-6-glucuronide (M6G), diamorphine, and normorphine. 35 he metabolites and part of intact morphine are eliminated by the kidney, requiring dose adjustment in renal failure due to the accumulation of M6G, a component ten times more potent than the original morphine. As such, avoid it in subjects with renal failure. 35

### Fentanil

Fentanil is a fat-soluble synthetic opioid with agonist activity at opioid receptors (μ). Its potency is approximately 80 times higher than morphine. It is available for intravenous, neuraxial, intramuscular, and transdermal administration. In the transdermal route, the peak of action occurs in 24 to 48 hours, and the total duration of effect lasts up to 72 hours when it requires change. As it has a slower titration, avoid it in acute postoperative pain needing faster dosage adjustment. 33

It is metabolized in the liver, generating inactive metabolites; therefore, it is safe for patients with renal failure and on dialysis. 35 In addition, avoid it in subjects with severe liver failure. 35

### Methadone

Methadone is a lipid-soluble synthetic opioid with a potency about ten times higher than morphine. Since it is an agonist for opioid receptors (μ) and an antagonist for N-methyl-D-aspartate (NMDA) receptors, methadone is a good alternative for treating neuropathic pain. It has good oral bioavailability and diffuse tissue distribution, explaining its long half-life of 8 to 59 hours. Its pharmacokinetics presents interindividual variation and erratic metabolism, with a variable conversion rate according to the dosage. Therefore, its management requires experience and care, with frequent assessment during increasing doses and longer intervals due to the risk of accumulation and respiratory depression. 25 29 Nausea is a warning sign of methadone intoxication. 36 In patients with heart disease, increased QT interval or previous long QT intervals warrant investigation because of the risk of arrhythmia, torsades de pointes, and sudden death. 25 29

Methadone metabolization occurs in the liver and intestine, and the bile eliminates its inactive metabolites. It is safe for patients with nephropathy, under dialysis, or with liver failure, but it requires careful monitoring of side effects. 29 36

### Oxycodone

Oxycodone is a semi-synthetic opioid with agonist activity at opioid receptors (μ). It is available in a controlled-release formulation for dosage every 12 hours. It has twice the analgesic potency of morphine. In a 20 mg dose per day, oxycodone is a weak opioid. In Brazil, there is no immediate-release oxycodone available, so morphine is the rescue medication. 37

Oxycodone metabolism occurs in the liver, and the drug depends on the kidney to excrete active metabolites and part of the intact compound. It is best to avoid it in patients with renal failure. 37

### Buprenorphine

Buprenorphine is an atypical strong opioid with partial agonist activity at opioid receptors (μ). It is 30 to 60 times more potent than morphine. The transdermal form, the only presentation available in Brazil, is a patch for application every 3 or 7 days. Patches are available in doses ranging from 5 to 40 mg. It has fewer side effects, and it is safe for older subjects. 38

### Tapentadol

Tapentadol is a strong atypical opioid with central action for pain relief. It has a dual mechanism of action: opioid receptor agonism (µ) and inhibition of norepinephrine reuptake in PHSC. Therefore, tapentadol offers an advantage over classic opioids in chronic pain control, either nociceptive or neuropathic. Compared to other opioids, tapentadol has fewer adverse effects, especially constipation. Tapentadol is the first-line treatment option for severe chronic low back pain with a neuropathic component. It is available in sustained-release presentations for administration every 12 hours in 50 to 250-mg doses. The maximum dosage reported in studies is 500 mg/day. 39

### Muscle Relaxing Agents

Several drugs act as central or peripheral muscle relaxants (including tizanidine, baclofen, benzodiazepines, carisoprodol, and cyclobenzaprine). Carisoprodol and cyclobenzaprine are the most prescribed muscle relaxant agents today. Carisoprodol metabolism gives rise to a toxic barbiturate known as meprobamate and, as a result, some countries have discontinued it. Cyclobenzaprine action is similar to tricyclic antidepressants, with equivalent indications and contraindications. There is evidence for its use in acute low back pain for a short period. 40 41

### Tricyclic and Dual Antidepressants and Gabapentinoid Anticonvulsant Agents

**Fig. 6:**
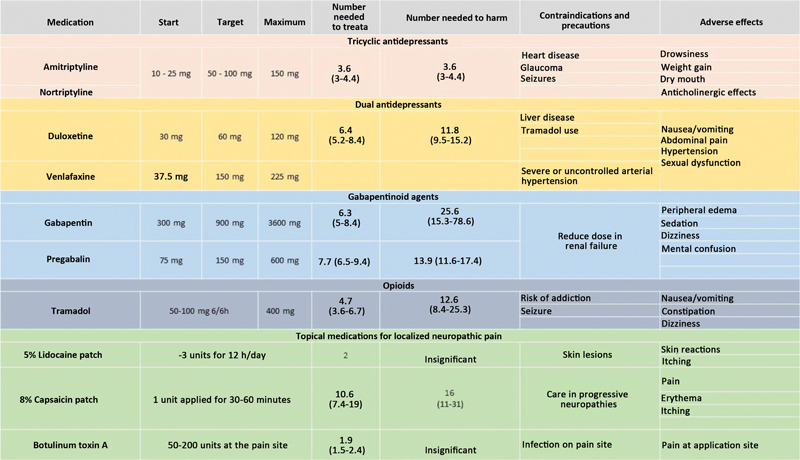
First- and second-line medications nociceptive pain treatment. ^a^ Number of treatments to achieve 50% pain relief with a 95% confidence interval (82-86).

The main NP guidelines indicate tricyclic antidepressants, dual antidepressants, gabapentinoid anticonvulsant agents, and 5% lidocaine patches as first-line treatments. It is worth noting that the analgesic effect of these antidepressants begins in 3 to 7 days. For these indications, such drugs cause no mood improvement, which requires higher doses of tricyclic agents and would have a later action. Fig. 6 illustrates the recommended dosage, most common adverse effects, number needed to treat (NNT), and number needed to harm (NNH) for first and second-line drugs to manage localized and systemic NP. 12 36 42 43 44

### Tricyclic Antidepressants

Tricyclic antidepressants (amitriptyline, imipramine, nortriptyline, etc.) are first-line drugs to treat neuropathic pain. 12 36

Amitriptyline inhibits several receptors, including muscarinic cholinergic, H1 histamine and alpha-adrenergic receptors, and norepinephrine and serotonin reuptake. Its side effects include xerostomia, weight gain, urinary retention, constipation, increased eye pressure, abnormalities in cardiac conduction, sedation, and orthostatic hypotension. Therefore, avoid amitriptyline in older patients. 45 Monitor its use in patients with angle-closure glaucoma due to the anticholinergic effects of tricyclic antidepressants. These drugs are contraindicated in the subjects with a left bundle branch block. 36

Nortriptyline predominantly inhibits the norepinephrine reuptake, causing fewer side effects than amitriptyline but with better tolerance. 36 It does not require dose adjustment in renal and hepatic insufficiency patients, and nortriptyline is preferable to amitriptyline. 36

### Dual Antidepressants

Dual antidepressants are first-line drugs for treating neuropathic pain. 12 36 42 43 44 Venlafaxine can increase blood pressure and cause hyponatremia. In doses lower than 150 mg/day, it acts as a selective serotonin reuptake inhibitor, but pain treatment requires a higher dosage. 36 Avoid duloxetine in patients with a glomerular filtration rate lower than 30 mL/min/1.73 m ^2^ and in those with a history of angle-closure glaucoma. Dual antidepressants are safer than tricyclic antidepressants in treating pain in older subjects. 36

### Gabapentinoid anticonvulsant agents

**Fig. 7:**
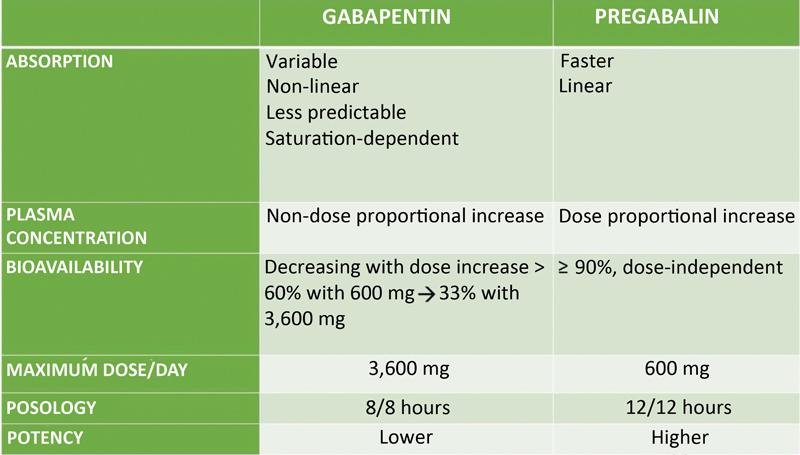
Differences between the gabapentinoid drugs available in Brazil. 47 56 57

Gabapentinoid anticonvulsant agents are first-line drugs for the treatment of neuropathic pain. 46 47 Gabapentin and pregabalin are gamma-aminobutyric acid (GABA) analogs but do not interact with this neurotransmitter. They bind to the alpha-2-delta subunits of calcium channels, blocking calcium entry into nerve endings and the release of excitatory neurotransmitters. 46 47 Pregabalin has a more linear and rapid absorption with good oral bioavailability. 46 47 These drugs may cause sedation, dizziness, and peripheral edema. 46 47 Patients with renal failure require dose adjustments. Fig. 7 shows the main differences between gabapentinoid agents. 36 46 47

### Topical medications

Topical medications play a significant role in nociceptive and neuropathic pain, as discussed below:

### 5% Lidocaine Patch

Topical lidocaine helps treat peripheral nociceptor sensitization and central nervous system hyperexcitability by blocking voltage-dependent sodium channels if used in recommended doses. Other effects on keratinocytes and immune cells or TRPV1 and TRPA1 receptor activation may contribute to the analgesic action of lidocaine. 45

It is a first-line medication for localized NP, such as postherpetic neuralgia and traumatic peripheral nerve injury. The lack of systemic side effects makes it a great option ( Table 3 ). 12 The patient uses a 5% lidocaine patch for 12 hours daily (maximum, three concomitant units) for two to four weeks when response reassessment occurs. 45 In addition to the local anesthetic effect, the patch protects against mechanical stimulation (dynamic allodynia), a common problem in neuropathic pain. It presents a good safety profile since only 3% (21 mg/unit) of the drug in each patch undergoes systemic absorption. 48 It also has a more favorable risk-benefit ratio than pregabalin (300 and 600 mg daily). 49 Data from a real-world study showed the efficacy and good tolerability of the 5% lidocaine patch, revealing that the treatment was significantly more effective when compared with first-line oral systemic medications. 49

**Table 3:** Main medications used in the treatment of acute and chronic nociceptive pain 24 49

		Initial dose	Posology	Therapeutic dose	Notes
Simple analgesic agents					
**Dipyrone (metimazole)**		500 mg to 1 g	6/6 hours	Up to 4 g/day	Agranulocytosis risk 24
**Paracetamol**		500 to 750 mg	6/6 hours	Up to 3 g/day	Hepatotoxicity 23
**Non-steroidal anti-inflammatory drugs: recommended for acute pain in minimal doses and for short courses**					
**Non-selective**		Variable	Variable	Variable	Gastrointestinal and renal effects 28
**Cox-2 selective**		Variable	Variable	Variable	Cardiovascular effects 32
**Opioid analgesic agent (per os)**					
**Weak**	Codeine	15 to 30 mg	Up to 6/6 hours	Variable Maximum dose: 360 mg/day	Dose-dependent: euphoria, nausea, obstipation, addiction, sedation, and respiratory depression 36
	Tramadol	50 to 100 mg	Up to 4/4 hours	Variable Maximum dose: 400 mg/day	
**Strong**	Morphine	5 to 30 mg	Up to 4/4 hours	Variable No maximum dose	
	Oxycodone	10 mg	12/12 hours	Variable No maximum dose	
	Methadone	2.5 to 5 mg	Up to 4/4 hours	Variable No maximum dose	
	Buprenorphine	5 to 40 mg	Every 3 or 7 days	Variable No maximum dose	
	Tapentadol	50 to 250 mg	12/12 hours	500 mg/day	
**Antispasmodic muscle relaxant agents**						
**Carisoprodol**	350 mg	Up to 6/6 hours	Maximum dose: 1.400 mg/day	Postural hypotension, drowsiness, and dizziness 45	
**Cyclobenzaprine**	5 mg a 10 mg	Up to 8/8 hours	20 to 40 mg Maximum dose: 60 mg/day	Drowsiness, dizziness, and dry mouth 46	

## Capsaicin

Capsaicin is the active compound from pepper, making it spicy. Its mechanism of action is via binding to nociceptors (sensory receptors sending signals for pain perception) in the skin and, specifically, to the TRPV1 receptor, which controls the flow of sodium and calcium ions across the cell membrane. Capsaicin binding opens the ion channel (influx of sodium and calcium ions), causing depolarization and the production of action potentials, perceived as itching, tingling, or burning sensations. In repeated applications or high concentrations, capsaicin has a long-lasting effect known as “desensitization,” probably due to a series of distinct actions that overload normal cell functions and can lead to the reversible degeneration of nerve terminals. Capsaicin is available in creams with low concentrations (0.025% and 0.075%). The guidelines for neuropathic pain management recommend the 8% capsaicin patch for treating localized neuropathic pain. 12 36

## Topical NSAIDs

Topical NSAIDs have good potential in acute pain, with acceptable NNT. NSAIDs are among the first-line treatment for chronic pain, including hand and knee osteoarthritis. Their prolonged use is safe because of their low systemic absorption. 50 51

## Non-pharmacological treatment

Non-pharmacological treatments comprise a diverse group of options to reduce pain intensity. Their primary goal is the functional improvement of the affected segment, promoting the quality of life and reintegrating the patient into social life. These treatments are frequently associated with pharmacological and interventional pain therapies within the context of a multimodal approach, seeking synergism of the positive effects of each technique. Physical activity is the leading non-pharmacological treatment, but the literature describes many others with a greater or lesser degree of evidence and good levels of biological plausibility. Non-pharmacological treatments are adjuvants in pain relief, mainly in chronic pain or patients using medications with intolerable adverse effects or contraindications. However, they require a way to interrupt pain or at least reduce it. 52

We must highlight that pain is multidimensional, requiring addressing within the biopsychosocial model. Further search for better evidence to support these practices and the development of new techniques for pain treatment are required. 52

### Interventional Pain Treatment

Pain refractory to conventional multimodal and interdisciplinary treatment may require interventional procedures for control. The interventional pain treatment is the fourth step of the adapted WHO analgesic ladder. 18 Some techniques are imaging-guided to reach a specific target (including nerves, intervertebral discs, and joints). These procedures may use chemicals (drugs), physical methods (radiofrequency), or biological products (platelet-rich plasma or concentrates of medicinal mesenchymal cells from bone marrow or fat), alone or in combination. 53 54 55 56 57

Blocks and infiltrations can be diagnostic or therapeutic. The advent of better imaging methods and the rise of ultrasonography (with no radiation and easily accessible) as a diagnostic tool to assist orthopedists in medical practice resulted in the potential performance of many of these blocks on an outpatient basis, helping pain control and bridging conservative and surgical treatment. These interventions are minimally invasive, present a low incidence of adverse effects, do not require hospital admission, and have satisfactory outcomes in pain control when properly indicated. In addition, in some cases, they can postpone and avoid surgeries. 53 54 55 56 57

The blocks can target the most varied structures of the human body, including the bursa, muscles, and joint sensory nerve endings and branches, such as the genicular branches of the knee in patients with osteoarthritis. 53 54 55 56 57

## Final considerations

The accurate diagnosis of the type of pain is paramount for its successful management Multimodal treatment, including pharmacological and non-pharmacological methods, is indicated for both acute and chronic pain to optimize analgesia and reduce adverse effects from using smaller doses of each medication. 36

The “start low and go slow” approach is essential for all medications to minimize adverse effects and increase treatment acceptance and adherence. It is critical to warn the patients of the risk of the most significant side effects, especially at the beginning of treatment. Most drugs, especially for NP treatment, act on the central nervous system, causing adverse events, particularly in the early titration phases. 36

To choose a treatment, consider the main therapeutic actions of each medication/intervention and their side effects. Selecting a medication within the first line requires conditioning the drug to the patient's demand. Remember that topical therapy can be an option due to its low side effect profile and effectiveness comparable to systemic drugs; a medication with sedative effects can help treat insomnia; and a drug with anxiolytic or non-sedating antidepressant action can help minimize the fatigue and lack of daytime motivation. 36

In acute or chronic pain refractory to conventional treatment, interventional procedures can help with analgesia. However, regardless of the selected treatment, the goal must be pain control to allow rehabilitation and improve function and quality of life. 36 Thus, aligning patients' expectations on treatment will lead to better outcomes.
