# Supplementary material for: Update on Musculoskeletal Pain Management
Source: Rev Bras Ortop (Sao Paulo). 2024 Feb 1;59(2):e160–71. doi: 10.1055/s-0043-1776135 (PMC11006528; doi:10.1055/s-0043-1776135)
Supplement: Related texts

# Research article: Atualização no manejo da dor musculoesquelética

**Keywords:** dor aguda, dor crônica, dor musculoesquelética, manejo da dor, dor pós-operatória

## Abstract

A dor é a queixa mais comum recebida pelo ortopedista no ambulatório e/ou emergência. Inúmeras publicações relatam o manejo inadequado tanto da dor aguda quanto da dor crônica pelos profissionais da saúde. O objetivo desse artigo de atualização é trazer informações sobre a dor musculoesquelética, sua classificação, avaliação, diagnóstico e abordagem terapêutica multimodal para cada situação. Desta maneira, nas dores agudas seu controle adequado possibilita um trabalho de reabilitação mais precoce, bem como diminui os índices de cronificação da dor. Nas dores crônicas sua abordagem além da diminuição de sua intensidade, visa também melhorar a qualidade de vida. Atualmente alguns procedimentos estão sendo cada vez mais utilizados com auxílio de aparato de imagem com objetivo diagnóstico e terapêutico.

## Introdução

Um em cada cinco adultos no mundo apresenta dor, sendo esta, a principal queixa que leva o indivíduo ao serviço médico tanto de nível ambulatorial, quanto emergencial, causando grande incapacidade ao indivíduo, bem como alto impacto econômico à sociedade. 1 2

De acordo com a IASP ( *International Association for the Study of Pain* ), a dor é uma experiência sensitiva e emocional desagradável, associada, ou semelhante àquela associada, a uma lesão tecidual real ou potencial, 3 podendo ser classificada conforme o tempo de evolução (aguda ou crônica) e quanto a sua fisiopatologia (nociceptiva, neuropática, nociplástica ou mista). Os princípios do tratamento da dor oncológica e não oncológica são diferentes. Sendo assim, o foco deste artigo será discutir sobre a avaliação e o manejo da dor musculoesquelética não oncológica.

## Fisiopatologia da dor

A dor é uma experiência percebida pelo sistema nervoso central e para que isso ocorra as informações devem chegar até o encéfalo através de vias e circuitos neuronais. Assim, a percepção da dor pelo córtex somatossensorial inicia-se na periferia, na transformação do estímulo doloroso de natureza mecânica, térmica e/ou química em sinal neuronal ( *transdução* ), que por sua vez é transmitida preferencialmente por fibras neurais do tipo A delta e C ( *transmissão* ), realizando a primeira conexão sináptica com neurônios localizados no corno posterior da medula espinhal (CPME). Nesta localização a informação sofre influência de estímulos inibitórios e excitatórios ( *modulação* ) provenientes do sistema nervoso periférico e central (Sistema Inibitório Descendente). 4

A partir do entendimento desta fisiopatologia podemos extrair a definição de que nocicepção é a percepção do estímulo doloroso que depende da integridade das vias citadas. Já a dor, diferente da nocicepção, é uma experiência complexa que envolve inúmeros fenômenos de modulação neuronal. 5

Muitas vezes os pacientes apresentam queixas dolorosas reais sem correlação com os exames complementares, apesar de não terem alterações estruturais nas imagens. A justificativa para suas queixas pode estar em um fenômeno conhecido como sensibilização, definida como aumento da excitabilidade da membrana celular dos neurônios sensitivos frente à estímulos dolorosos, podendo ser periférica (terminação nervosa), ou central (CPME e todo neuro-eixo). 6

## Epidemiologia

As taxas de prevalência de dor crônica variam entre 11% e 40% na população americana (média de 20,4%), 43,5% no Reino Unido com taxa de dor moderada a grave incapacitante variando de 10,4% a 14,3%. Dados brasileiros apontam que a prevalência de dor crônica variou de 23,02 a 76,17% (média: 45,59%), afetando mais o sexo feminino. A região do Brasil com maior prevalência foi a região centro-oeste (56,25%). Quanto às classificações de mecanismos da IASP, a dor possivelmente nociceptiva obteve prevalência de 36,70%, já a neuropática foi de 14,5% e a dor nociplástica de 12,5%. 7

## Classificação da dor

A classificação correta do tipo de dor é essencial para a escolha adequada de seu tratamento, visto que cada tipo de dor tem um tratamento distinto. 8 Para melhorar a interpretação das várias síndromes de dor crônica a IASP reformulou a classificação internacional de doenças (CID-11) para favorecer as pesquisas e diagnósticos. Nas Fig. 1 e 2 são mostradas a nova divisão. 9

**Fig. 1:** Classificação CID-11 para dor crônica. 11

**Fig. 2:** Subdivisão das síndromes dolorosas crônicas de acordo com o CID-11. 11

## Quanto ao tempo de evolução

A dor aguda é uma resposta fisiológica normal do organismo frente ao estímulo doloroso, caracterizada por condição autolimitada e de curta duração, geralmente menor de 3 meses e que cessa ao término da cicatrização e reparação dos tecidos acometidos. 8 10

A dor crônica é considerada como aquela que persiste além do tempo normal de cicatrização ou que ultrapasse três meses de duração, com todas as repercussões que nela podem estar acopladas.

## Quanto ao tipo de dor

- Dor nociceptiva: que surge de danos reais ou potenciais a tecidos não-neurais. 11 Está presente nas dores traumáticas agudas e no pós-operatório, e as classes de medicamentos mais utilizada são analgésicos simples e opioides, os anti-inflamatórios não hormonais (AINH) e relaxantes musculares ( Tabela 1 ). 6
- Dor neuropática (DN) é aquela decorrente de lesão ou disfunção de estruturas do sistema nervoso periférico ou central, como ocorre na hérnia de disco com compressão radicular e na síndrome do túnel do carpo ( Tabela 2 ). 12 A manifestação clínica ocorre com dor em queimação, frio doloroso, choque elétrico, coceira, alfinetada e agulhada. Além disso, alterações no exame neurológico podem ser identificadas como hiperalgesia, hiperpatia e alodinia. Nesta condição utilizamos primariamente os medicamentos adjuvantes (antidepressivos tricíclicos, antidepressivos duais e anticonvulsivantes gabapentinóides) e os opioides são reservados para analgesia no início do tratamento até o ajuste da dose do adjuvante e para permitir a reabilitação quando a analgesia é insuficiente. 8 Vide Fig. 3 12
- Dor Nociplástica: mais recentemente descrita é aquela que não está associada a nenhuma lesão tecidual (neural ou não). Como exemplos desse tipo de dor temos a enxaqueca e fibromialgia, e o seu tratamento é baseado em tratamento multimodal e interdisciplinar. 12
- Dor mista é a concomitância dos tipos de dor acima descritos em um único indivíduo. 4

**Tabela 1:** Principais medicamentos recomendados para o tratamento de dor nociceptiva em adultos

		Dose inicial	Posologia	Dose terapêutica	Observações
**Analgésicos simples**					
Dipirona		500 mg-1g	6/6h	até 4g/dia	Risco de agranulocitose 24
Paracetamol		500-750 mg	6/6h	até 3g/dia	Hepatotoxicidade 23
**AINH: recomenda-se o uso para dores agudas com a mínima dose e curto período**					
Não seletivos		Variável	Variável	Variável	Gastrointestinal e renal 28
Cox-2 seletivo		Variável	Variável	Variável	Cardiovasculares 32
***Analgésico opioide (via oral)***					
Fraco	Codeina	15 a 30 mg	Até 6/6h	Variável. Máx 360 mg/dia	Dose-dependente: euforia, náuseas, obstipação, adição, sedação e depressão respiratória. 36
	Tramadol	50–100 mg	Até 4/4h	Variável. Máx 400 mg/dia	
Forte	Morfina	5 a 30 mg	Até 4/4h	Variável. Sem dose máxima	
	Oxicodona	10 mg	12/12h	Variável. Sem dose máxima	
	Metadona	2,5 a 5mg	Até 4/4h	Variável. Sem dose máxima	
	Buprenorfina	5mg	A cada 7 dias	Variável. Sem dose máxima	
**Relaxantes musculares antiespasmódicos**					
Carisoprodol		350mg	Até 6/6h	Máx 1400 mg/d	Hipotensão postural, sonolência; tontura. 45
Ciclobenzaprina		5mg a 10 mg	Até 8/8h	20 a 40 mg Máx 60mg/d	sonolência, tontura e boca seca 46

**Tabela 2:** Medicamentos considerados primeira linha para o tratamento de dor neuropática pelas principais diretrizes 49

		Dose inicial	Posologia	Dose terapêutica	Efeitos adversos
***Antidepressivos***					
Tricíclicos	Amitriptilina Imipramina Nortriptilina	10 a 25mg	1x dia, 3 horas antes de dormir	25 a 150mg/dia	Sonolência, tonturas, tremores, dores de cabeça, hipotensão postural, boca seca, náuseas, constipação, ganho de peso, diminuição da libido, hiperidrose, retenção urinária e ideação suicida. 47
Duais	Duloxetina	30mg	1x dia pela manhã	60 a 120mg/dia	Náusea, boca seca, sonolência, aumento da pressão arterial e ideação suicida 48
	Venlafaxina	37,5 a 75mg	1x dia pela manhã	150 a 225mg/dia	
***Anticonvulsivantes***					
Gabapentina (GBP)		300mg	8/8 h	1200 a 3600mg/dia	Tontura, náusea, sonolência, edema de extremidades, ganho ponderal, visão turva e ideação suicida 48
Pregabalina (PGL)		50 a 75mg	12/12h	150 a 600 mg/dia	
*Tópicos*					
Patch de lidocaína 5%		1 a 3 patchs	12h sem/12h com o path	1 a 3 patchs	

**Fig. 3:** Guideline de recomendações francesas de 2020 para o tratamento da dor neuropática. 17

## Avaliação e diagnóstico da dor

A avaliação do indivíduo deve ser feita de maneira completa, seguindo as etapas da anamnese, exame físico e exames subsidiários. Aqui é importante descrever quando, como e os fatores de melhora e de piora e quais foram as tentativas de tratamento pregressas que deram mais ou menos resultado e, tão importante quanto a descrição é a a real definição se há ou não correlação clínico radiológica, para que ao término da avaliação possa ser estabelecido uma relação nexo causal da dor ou não, uma vez que, são pacientes, no caso da dor crônica, que já experimentaram muitos tratamentos e diversas dimensões da vida estão afetadas (qualidade de vida, funcionalidade, relações sociais, etc.). Escalas podem facilitar a mensuração subjetiva da dor (escala verbal numérica e escala verbal analógica (EVN,EVA).Ferramentas de rastreio de dor neuropatia também são uteis, como O DN4, LANSS, e o *painDetect* . Ao término da avaliação deve ficar claro quais os principais mecanismos envolvidos na dor e se há ou não relação nexo causal com a dor. 13 14 15 16

## Tratamento

## Tratamento Farmacológico

Na dor aguda, o diagnóstico e tratamento específico da causa da dor é essencial, uma vez que ela tem função fisiológica de alertar alguma lesão ou doença ativa. Para tratar sintomaticamente a dor, adotamos a escada analgésica da Organização Mundial de Saúde (OMS) 17 orientado pela intensidade da dor (EVA ou EVN). O primeiro degrau da escada analgésica da OMS ( Fig. 4 ) consiste na dor leve, sendo indicado uso de analgésicos simples associado a um AINH conforme as contraindicações de seu uso. No segundo degrau (dores moderadas) pode sem utilizados opioides fracos, no terceiro degrau (dores fortes), pode-se utilizar opioides fortes. Existe uma adaptação da escada analgésica da OMS adicionando um quarto degrau, consistindo em procedimentos intervencionistas para a dor. 18

**Fig. 4:** Adaptação da escada analgésica da OMS, baseada na analgesia por degraus. As medicações do primeiro degrau são indicadas para dor leve, do segundo para dor moderada e o terceiro para dor intensa a forte. Posteriormente o quarto degrau foi adicionado a escada original para abordar as dores refratárias ao tratamento medicamentoso. 22 23

A evolução da dor aguda para crônica está associada à vários fatores, como a intensidade da dor, aspectos sociais e psicológicos e até genéticos, contudo, o tratamento apropriado da dor aguda é fundamental para evitar a sua progressão. No contexto ortopédico, o tipo de anestesia, e os fármacos utilizados no perioperatório podem diminuir o risco de cronificação da dor. 8 A falha na formação médica em dor ainda é uma das principais causas de manejo inadequado da dor. 8

Toda dor crônica deve ser tratada de forma multimodal, tanto do ponto de vista farmacológico, quanto não farmacológico., valendo-se de mecanismos de ação distintos com potenciais sinérgicos. 19

## Analgésicos Simples

A dipirona e o paracetamol são os mais utilizados na prática clínica. Ambos são analgésicos e antipiréticos com eficácias semelhantes e pouca atividade anti-inflamatória e inibitória sobre COX-1 e COX-2. 20 21 Apresentam perfis de efeitos colaterais distintos. O paracetamol pode ser hepatotóxico em doses altas, sendo considerado a principal causa de falência hepática aguda nos EUA. 22 Mas é seguro numa dosagem de até 4g/dia. A dipirona por sua vez pode apresentar como efeito adverso a agranulocitose, contudo a incidência na América Latina é muito baixa. 23 Ambos podem ser utilizados nas dores agudas e crônicas e não necessitam de ajuste de dose na insuficiência renal. 24

## Anti-inflamatórios não Hormonais

Os AINHs estão indicados em casos de dor aguda, nas agudizações de dores crônicas e dores inflamatórias de causas nociceptivas. A recomendação atual é usar a menor dose possível por poucos dias devido ao risco dos efeitos colaterais, principalmente no uso prolongado. 25

Sua ação consiste no bloqueio da síntese de prostaglandinas por inibição das enzimas ciclo-oxigenases tipo 1 (COX-1), constitutiva, cuja inibição gera efeitos adversos gastrointestinais e a ciclo-oxigenases tipo 2 (COX-2), induzida nos processos inflamatórios. 26 Quanto menor. Seletividade para COX2, maior o risco de evento adverso gastrointestinal e de sangramentos, e quanto maior a seletividade COX2, maior a chance de efeito cardiovasculares. Os AINHs são os medicamentos mais utilizados no tratamento para dor crônica, apesar da não serem recomendados para esta indicação. São contraindicados na insuficiência renal e hepática. 26

## Analgésico Opioides

Os opioides podem ser típicos e atipicos, fracos ou fortes ( Fig. 5 ). Os opioides fracos são utilizados em dores agudas de intensidades moderada ou forte, e podem ser utilizados na dor crônica em quadros de exacerbação. Quanto aos opioides fortes, estes têm indicação precisa nas dores agudas de forte intensidade, e eventualmente em casos de díficil controle. 27

**Fig. 5:** Classificação dos opioides quanto à potência e mecanismo de ação. 35 36 37 38 39 40 41 49

Apesar do receio por parte dos ortopedistas, o uso racional de opioides, torna a prescrição segura. Titulação adequada, pesquisa do risco de adição e uso pelo menor tempo possível, até que medicações adjuvantes ou tratamento específicos surjam efeito desejado, compõem a boa prática. 28

De forma prática é recomendada o uso de um opioide de curta duração como a morfina para titulação da menor dose efetiva com aumentos sucessivos até obter o alívio da dor. A partir dessa dose, cálculos para rotação ou até mesmo cálculo de dose de resgate podem ser ajustadas. Para o resgate utiliza-se 1/10 a 1/6 da dose diária total, caso haja um escape de dor entre os intervalos, assim como a dose para apresentação de liberação controlada e a dose equianalgésica para rotação de opioides. Existem variadas tabelas de conversão empíricas que servem de orientação. Tolera6ncia cruzada, hiperalgesia induzida por opiodes, ou efeitos intoleráveis são critérios para a rotação de opioides. 29

Outros efeitos colaterais dos opioides são: constipação, náuseas, vômitos, hiperalgesia, tolerância, abstinência, entre outros. Destas, a constipação é a que não sofre tolerância, devendo ser abordada de forma profilática, visto que aumenta morbidade e mortalidade com piora da qualidade de vida. Pode ser tratada com laxantes, como lactulose, bisacoldil, sena, polietilenoglicol, associado a mudança alimentar e hidratação. Cuidado especial deve ser dado aos idosos que recenem oipioides, pelo risco aumentado de efeitos adversos e quedas. 30

Para que não ocorra no Brasil, o que se sucedeu nos Estados Unidos, a educação médica continuada desse ser a chave do processo. 31

## Tramadol

Opioide fraco com atividade agonista em receptores opioides (μ) e ação dual por meio da inibição sobre a recaptação de serotonina e noradrenalina sendo vantajoso no tratamento de dores nociceptivas e neuropáticas. 32

Apresenta como desvantagem a diminuição do limiar convulsivo. O uso concomitante com antidepressivos, principalmente os seletivos da recaptação de serotonina e os duais, podem causar síndrome serotoninérgica. Causa menos constipação que a codeína.

É necessário ajuste de dose na insuficiência renal avançada, sendo dialisável e seguro para os pacientes dialíticos. 32

## Codeína

Derivado da morfina é metabolizada no fígado em codeína-6-glicuronídeo e desmetilada em morfina, formando metabólitos ativos que agem em receptores opioides (μ). 33 Alguns pacientes não apresentam a enzima de conversão da codeína, sendo insensíveis a medicação. Apresenta eliminação renal, sendo necessário cuidados no paciente com insuficiência renal. Como não é facilmente removida na diálise, evitar em pacientes dialíticos. 34

## Morfina

Opioide natural com atividade agonista em receptores opioides (μ) usada como referência para titulação e cálculo de doses equianalgésicas para rotação de opioides. Disponível em apresentação de liberação imediata e controlada.

Sofre metabolização hepática em morfina-3-glicuronídeo, morfina-6-glicuronídeo (M6G), diamorfina e normorfina. 35 Os metabólitos e parte da morfina são eliminados pelo rim, sendo necessário ajuste de dose na insuficiência renal, pelo acúmulo do M6G, componente dez vezes mais potente que a morfina original. Deve ser evitada na insuficiência renal. 35

## Fentanil

Opioide sintético com atividade agonista em receptores opioides (μ), lipossolúvel, com potência de cerca de 80 vezes mais que a morfina. Pode ser utilizada por via, endovenosa, neuroeixo, intramuscular e transdérmica. Esta última via apresenta pico de ação em 24 a 48h e duração total do efeito até 72h, quando deve ser trocado. Por ser de titulação mais lenta não deve ser usada na dor aguda pós-operatória que requer um ajuste mais rápido da dosagem. 33

É metabolizada no fígado, gerando metabólitos inativos, sendo um fármaco seguro para uso em portadores de insuficiência renal e dialíticos. 35 Na insuficiência hepática grave deve ser evitado. 35

## Metadona

Opioide sintético lipossolúvel com potência cerca de 10 vez maior que a morfina. É agonista em receptores opioides (μ) e antagonista em receptores N-metil-D-aspartato (NMDA), sendo uma boa alternativa para o tratamento da dor neuropática. Apresenta boa biodisponibilidade oral com difusa distribuição tecidual, explicando a sua meia-vida longa de 8 a 59 horas. Apresenta uma farmacocinética com variação interindividual e metabolização errátil, com taxa de conversão variável de acordo com a dosagem, sendo que seu manejo exige experiência e cuidados, com avaliação frequente durante o aumento das doses e com intervalos maiores pelo risco de acúmulo e depressão respiratória. 25 29 A náusea é um sinal de alerta para intoxicação pela metadona. 36 Para pacientes com doenças cardíacas deve-se pesquisar aumento do intervalo QT ou QT longo prévio pelo risco de arritmia, *torsades de pointes* e morte súbita. 25 29

É metabolizada no fígado e no intestino, com formação de metabólitos inativos eliminados através da bile. É seguro para nefropatas e dialíticos e pacientes com insuficiência hepática, mas necessita monitorização cuidadosa dos efeitos colaterais. 29 36

## Oxicodona

Opioide semissintético com atividade agonista em receptores opioides (μ) apresentando formulação de liberação controlada como posologia a cada 12h. Apresenta o dobro da potência analgésica da morfina. Na dose de 20mg por dia é considerada opioide fraco. No Brasil não há apresentação de oxicodona de liberação imediata, sendo necessário utilizar a morfina como medicação para resgate. 37

Sua metabolização é hepática e o fármaco depende do rim para excreção de metabólitos ativos e parte do composto original. A oxicodona deve ser evitada na insuficiência renal. 37

## Buprenorfina

Opioide forte atípico com atividade agonista parcial em receptores opioides (μ), 30 a 60 vezes mais potente que a morfina. A forma transdérmica, única apresentação no mercado brasileiro, tem disponibilidade de posologia do adesivo para aplicação a cada 3 ou 7 dias. Os adesivos estão disponíveis nas doses de 5 a 40 mg. Apresenta menos efeitos colaterais, sendo segura para uso nos idosos. 38

## Tapentadol

Tapentadol é um opioide atípico forte que atua centralmente para atenuar a dor. Apresenta um mecanismo duplo de ação: um agonismo do receptor opioide (µ) e inibição da recaptação de noradrenalina no CPME. Diante disso, o tapentadol oferece uma vantagem sobre os opioides clássicos no controle da dor crônica, desde nociceptiva até neuropática. Comparado a outros opioides, o tapentadol está associado a menos efeitos adversos, especialmente constipação intestinal. O tapentadol pode ser considerado uma opção de tratamento de primeira linha no tratamento da dor lombar crônica grave com componente neuropático. Tem apresentações de liberação programada para uso de 12 em 12 horas, nas doses de 50 até 250 mg. A dose máxima em estudos é de 500 mg/dia. 39

## Relaxantes musculares

Existem inúmeros fármacos que atuam como relaxante muscular (tizanidina, baclofeno, benzodiazepínicos, carisoprodol e ciclobenzaprina), tanto de maneira central quanto periférica. Atualmente o carisoprodol e a ciclobenzaprina são os mais prescritos. O carisoprodol é metabolizado e dá origem a um barbitúrico conhecido como meprobamato, tóxico, por essa razão alguns países descontinuaram o uso deste fármaco. A ciclobenzaprina possui ação similar ao dos antidepressivos tricíclicos, sendo suas indicações e contra indicações semelhantes. Há evidência para uso em dor lombar aguda por um período curto. 40 41

## Antidepressivos tricíclicos e duais e anticonvulsivantes gabapentinoides

As principais diretrizes de DN indicam como primeira linha de tratamento os antidepressivos tricíclicos, os antidepressivos duais, os anticonvulsivantes gabapentinoides e o emplastro de lidocaína 5%. É importante ressaltar que o efeito analgésico destes antidepressivos inicia-se por volta de 3 a 7 dias sendo dissociado do efeito de melhora de humor, em que as doses são maiores, no caso de tricíclicos, e ação mais tardia. A Fig. 6 ilustra a posologia recomendada, efeitos adversos mais comuns, número necessário para tratar (NNT) e número necessário para causar danos (NNH) para as drogas de primeira e segunda linha do manejo da DN localizada e sistêmica. 12 36 42 43 44

**Fig. 6:** Medicações de primeira e segunda linhas para o tratamento da DN. ^a^ Número de tratamentos para conseguir 50% de alívio da dor com intervalo de confiança de 95% (82-86)

## Antidepressivos Tricíclicos

Os antidepressivos tricíclicos (amitriptilina, imipramina, nortriptilina, etc)são considerados fármacos de primeira linha para o tratamento da dor neuropática. 12 36

A amitriptilina age inibindo vários receptores, dentre eles colinérgico muscarínico, histamínico H1, alfa-adrenérgico, recaptação de noradrenalina e serotonina, entre outros, podendo causar vários efeitos adversos como xerostomia, ganho de peso, retenção urinária, constipação, aumento de pressão ocular, anormalidades na condução cardíaca, sedação e hipotensão ortostática, devendo ser evitado nos idosos. 45 Deve ter seu uso monitorizado em pacientes portador de glaucoma de ângulo fechado pelos efeitos anticolinérgicos dos tricíclicos. São contraindicados em caso de presença de bloqueio de ramo esquerdo. 36

A nortriptilina inibe a recaptação predominantemente da noradrenalina, causando menos efeitos colaterais que a amitriptilina, com melhor tolerância. 36 Não necessita de ajuste da dose na insuficiência renal e hepática, sendo preferível do que amitriptilina. 36

## Antidepressivos Duais

Os antidepressivos duais são considerados fármacos de primeira linha para o tratamento da dor neuropática. 12 36 42 43 44 A venlafaxina pode causar aumento de PA e hiponatremia. Abaixo de 150mg/dia funciona como inibidor seletivo da recaptação de serotonina, sendo que tratamento da dor é necessário atingir dose maior. 36 A duloxetina deve ser evitada em paciente com TFG menor que 30mL/min/1,73m ^2^ e em pacientes com história de glaucoma de ângulo fechado. Os duais são mais seguros que os tricíclicos no tratamento da dor no idoso. 36

## Anticonvulsivantes Gabapentinoides

Os anticonvulsivantes gabapentinoides são considerados fármacos de primeira linha para o tratamento da dor neuropática. 46 47 Gabapentina e pregabalina são análogos do ácido gama-aminobutírico (GABA), sem interação com este neurotransmissor. Atua por meio da ligação às subunidades alfa-2-delta de canais de cálcio, bloqueando a entrada de cálcio nas terminações nervosas e a liberação de neurotransmissores excitatórios. 46 47 A pregabalina apresenta uma absorção mais linear e rápida com boa biodisponibilidade oral. 46 47 Podem causar sedação, tontura, edema periférico. 46 47 É preciso de ajuste na dose na insuficiência renal. As principais diferenças entre os gabapentinoides são discriminadas na Fig. 7 . 36 46 47

**Fig. 7:** Diferenças entre as drogas gabapentinoides disponíveis no Brasil. 47 56 57

## Medicações Tópicas

As medicações tópicas possuem um papel importante nas dores nociceptivas e nas dores neuropáticas, como veremos a seguir:

## Emplastro de Lidocaína 5%

A lidocaína tópica auxilia no tratamento da sensibilização dos nociceptores periféricos e a hiperexcitabilidade do sistema nervoso central, via bloqueio dos canais de sódio voltagem dependente, se usada nas doses recomendadas. Outros efeitos nos queratinócitos e células imunes, ou ativação de receptores (TRPV1 e TRPA1), podem contribuir para o efeito analgésico da lidocaína. 45

É medicação de primeira linha da DN localizada, como neuralgia pós-herpética e lesão traumática de nervo periférico, onde a falta de efeitos colaterais sistêmicos a torna uma ótima opção ( Tabela 3 ). 12 O adesivo, emplastro de lidocaína 5%, deve ser utilizado por 12 horas, por dia, no máximo 3 unidades concomitantes, e por um período entre 2 a 4 semanas para que a resposta seja avaliada. 45 Além do efeito anestésico local, o adesivo fornece proteção contra estimulação mecânica (alodinia dinâmica), que é um problema frequente na dor neuropática. Apresenta grande segurança, pois somente 3% (21 mg/por unidade) da droga contida em cada emplastro sofre absorção sistêmica 48 e uma relação risco-benefício mais favorável em comparação com a pregabalina (300 e 600 mg diários). 49 Em dados de estudos em mundo real a eficácia e a boa tolerabilidade do emplastro de lidocaína 5% foi observada, evidenciando que o tratamento foi significativamente mais eficaz quando comparado com medicamentos sistêmicos orais de primeira linha. 49

**Tabela 3:** Principais medicamentos utilizados no tratamento da dor nociceptiva aguda e crônica 24 49

		Dose inicial	Posologia	Dose terapêutica	Observações
**Analgésicos simples**					
**Dipirona**		500 mg-1g	6/6h	até 4g/dia	Risco de agranulocitose 24
**Paracetamol**		500-750 mg	6/6h	até 3g/dia	Hepatotoxicidabe 23
**AINH: recomenda-se o uso para dores agudas com a mínima dose e curto período**					
**Não seletivos**		Variável	Variável	Variável	Gastrointestinal e renal 28
**Cox-2 seletivo**		Variável	Variável	Variável	Cardiovasculares 32
**Analgésico opioide (via oral)**					
**Fraco**	Codeina	15 a 30 mg	Até 6/6h	Variável. Máx 360 mg/dia	Dose-dependente: euforia, náuseas, obstipação, adição, sedação e depressão respiratória 36 .
	Tramadol	50–100 mg	Até 4/4h	Variável. Máx 400 mg/dia	
**Forte**	Morfina	5 a 30 mg	Até 4/4h	Variável. Sem dose máxima	
	Oxicodona	10 mg	12/12h	Variável. Sem dose máxima	
	Metadona	2,5 a 5mg	Até 4/4h	Variável. Sem dose máxima	
	Buprenorfina	5-40mg	A cada 3 ou 7 dias	Variável. Sem dose máxima	
	Tapentadol	50-250mg	12/12h	500 mg/dia	
**Relaxantes musculares antiespasmódicos**					
**Carisoprodol**	350mg	Até 6/6h	Máx 1400 mg/d	Hipotensão postural, sonolência; tontura 45 .	
**Ciclobenzaprina**	5mg a 10 mg	Até 8/8h	20 a 40 mg Máx 60mg/d	sonolência, tontura e boca seca 46	

## Capsaicina

A capsaicina é o composto ativo das pimentas, responsável por torná-las picantes. Seu mecanismo de ação é via ligação a nociceptores (receptores sensoriais responsáveis por enviar sinais que causam a percepção da dor) na pele e, especificamente, ao receptor TRPV1, que controla o fluxo de íons de sódio e cálcio através da membrana celular. A ligação abre o canal iônico (influxo de íons sódio e cálcio), causando despolarização e produção de potenciais de ação, que geralmente são percebidos como sensações de coceira, formigamento ou queimação. Aplicações repetidas ou altas concentrações dão origem a um efeito duradouro, que tem sido denominado “dessensibilização”, provavelmente devido a uma série de efeitos distintos que juntos sobrecarregam as funções normais da célula e podem levar à degeneração reversível dos terminais nervosos. Atualmente temos cremes com baixas concentrações (0,025% e 0,075%). Nas diretrizes de manejo da dor neuropatia a apresentação em emplastro a 8% figura nas diretrizes de manejo da dor neuropatica localizada. 12 36

## AINHs tópicos

Os AINH tópicos apresentam um bom potencial de uso na dor aguda, com bom NNT e na dor crônica figuram entre a primeira linha de tratamento na osteoartite de mãos e joelhos, cm boa segurança para uso por tempo prolongado, devido a baixa absorção sistêmica do produto. 50 51

## Não Farmacológicos

Este é um grupo diversificado de opções terapêuticas que visa a diminuição da intensidade da dor, como objetivo primordial de melhorar a função do segmento acometido, promover a qualidade de vida do paciente em sofrimento e reintegrar ao convívio social. São tratamentos frequentemente utilizados em associação ao tratamento farmacológico e intervencionista da dor, dentro do contexto do tratamento multimodal, buscando sinergismo dos efeitos positivos de cada técnica. Seguramente a atividade física seja o principal tratamento não farmacológico, mas muitos outros estão descritos na literatura, com maior ou menor grau de evidência, e com bons níveis de plausibilidade biológica. São utilizadas como adjuvantes no alívio da dor, principalmente na dor crônica ou para aqueles pacientes em uso de medicações cujo os efeitos adversos são intoleráveis ou são contra-indicados, mas necessitam de uma forma de interrupção de dor ou pelo menos redução. 52

Devemos ressaltar que a dor é multidimensional e deve ser abordada dentro do modelo biopsicossocial. Mas também é real e necessária a busca de melhores evidências para embasar estas práticas, assim como novas técnicas, no tratamento da dor. 52

## Tratamento Intervencionista da dor

Em casos refratários ao tratamento multimodal e interdisciplinar convencional, procedimentos intervencionistas podem ser indicados para o controle da dor, sendo considerado o quarto degrau da Escada Analgésica da OMS adaptada. 18 Para isso, algumas técnicas podem ser guiadas por meio de imagem, a fim de alcançar um alvo específico (nervo, disco intervertebral, articulação). Nestes procedimentos podem ser utilizados meios químicos (drogas), meios físicos (radiofrequência), e/ou produtos biológicos (plasma rico em plaquetas ou concentrados de células medicinais mesenquimais da medula óssea ou da gordura). 53 54 55 56 57

Os bloqueios e infiltrações podem ser diagnósticos ou terapêuticos. Com o advento de melhores métodos de imagem e com a ascensão da ultrassonografia (sem uso de radiação e de fácil acesso) como ferramenta diagnóstica e auxiliar do ortopedista na prática médica muitos desses bloqueios podem ser feitos de maneira ambulatorial, auxiliando o paciente no controle da dor, na ponte entre o tratamento conservador e cirúrgico. São intervenções minimamente invasivas, com incidência de efeitos adversos baixa, sem necessidade de internação hospitalar, e resultados satisfatórios no controle da dor quando bem indicados, sendo que em algumas situações podem postergar e evitar cirurgias. 53 54 55 56 57

Os bloqueios podem ser voltados para as mais variadas estruturas do corpo humano, desde a bursa, o músculo, até terminações e ramos nervosos sensitivos articulares, como por exemplo os ramos geniculares do joelho, em casos de osteartite. 53 54 55 56 57

## Considerações Finais

Para o sucesso do manejo da dor o diagnóstico preciso do tipo de dor é essencial. O tratamento multimodal, através de medidas farmacológicas e não farmacológicas, está indicado tanto para as dores agudas quanto para as dores crônicas, o objetivo é otimizar a analgesia e diminuir os efeitos adversos com o uso de doses menores de cada medicamento. 36

A abordagem “começar baixo e ir devagar” é essencial para todos os medicamentos para minimizar os efeitos adversos e aumentar aceitação e adesão ao tratamento, sempre alertando os pacientes sobre o risco dos efeitos colaterais mais importantes, principalmente no início da administração. A maioria das drogas, especialmente para o tratamento da DN, tem efeitos no sistema nervoso central, consequentemente ocasionando eventos adversos especialmente nas fases iniciais de titulação. 36

Na escolha terapêutica deve-se levar em consideração as principais ações terapêuticas de cada medicamento/intervenção bem como seus efeitos colaterais. Escolher uma medicação dentro das de primeira linha, requer condicionar a droga à demanda do paciente. Lembrar que a terapia tópica pode ser uma escolha devido seu baixo perfil de efeitos colaterais e efetividade equiparada a drogas sistêmicas; um medicamento com efeitos sedativos pode ajudar a tratar a insônia em pacientes com dor; e uma medicação com ação ansiolítica ou antidepressiva não sedativa pode auxiliar a minimizar efeitos de fadiga e falta de motivação diurna. 36

Em casos refratários ao tratamento convencional, independente da dor estar na fase aguda ou crônica, os procedimentos intervencionistas em dor podem auxiliar na analgesia. Contudo, independentemente do tratamento escolhido o objetivo deve ser controlar a dor para permitir a reabilitação, melhorar a função e a qualidade de vida dos pacientes. 36 Assim alinhar com os pacientes suas expectativas frente ao tratamento os conduzirá a melhores resultados.
